# Investigating the Impact of Pressure Relief Performance on the Occurrence of Pressure Injuries and Shoulder Pain in Wheelchair Users with Spinal Cord Injury (PRperf Study): Study Protocol for a Prospective Observational Study

**DOI:** 10.3390/mps8030062

**Published:** 2025-06-06

**Authors:** Yannik Schürch, Anneke Hertig-Godeschalk, Inge Eriks-Hoogland, Anke Scheel-Sailer, Martin W. G. Brinkhof, Ursina Arnet

**Affiliations:** 1 Swiss Paraplegic Research, 6207 Nottwil, Switzerland; yannik.schuerch@paraplegie.ch (Y.S.); anneke.hertig@paraplegie.ch (A.H.-G.); inge.eriks@paraplegie.ch (I.E.-H.); anke.scheel-sailer@paraplegie.ch (A.S.-S.); martin.brinkhof@paraplegie.ch (M.W.G.B.); 2 Faculty of Health Sciences and Medicine, University of Lucerne, 6005 Lucerne, Switzerland; 3 Swiss Paraplegic Centre, 6207 Nottwil, Switzerland; 4 Centre for Rehabilitation and Sports Medicine, Insel Group, University Hospital, University of Bern, 3010 Bern, Switzerland

**Keywords:** spinal cord injury, wheelchair, pressure injury, shoulder pain, prevention, longitudinal study

## Abstract

Background: Pressure injuries (PIs) and shoulder pain (SP) are frequent problems in individuals with spinal cord injury (SCI), affecting both quality of life and healthcare use. Although pressure relief (PR) is recommended to prevent PIs, it is often not performed regularly, and its long-term benefits remain unclear. Furthermore, some PR methods may contribute to SP, resulting in conflicting clinical guidelines. This study aims to objectively measure PR performance and investigate its long-term relationship with PI and SP. Methods: This study is a longitudinal observational study involving 70 manual wheelchair users with complete SCI. Over one year, participants attend five study visits to assess confounding factors such as comorbidities and shoulder range of motion. PR performance (technique, frequency, duration) is continuously monitored for three weeks after each of the first four visits using textile measurement mats, while SP is assessed weekly with a questionnaire. Causal associations with PI and SP will be examined using directed acyclic graphs and multivariable regression modelling. Results: The study is ongoing. Long-term objective data on PR performance will provide insights into its relationship with PI and SP. Conclusions: Findings will inform clinical practice and contribute to improved evidence-based PR guidelines for individuals with SCI.

## 1. Introduction

Pressure injuries (PIs) and shoulder pain (SP) are two highly prevalent and consequential secondary health conditions among individuals with spinal cord injury (SCI) who rely on a wheelchair for mobility and activities of daily living [1,2,3,4,5,6]. A PI is a localised damage to the skin and underlying soft tissue caused by intense and/or prolonged pressure or pressure in combination with shear [7,8]. Prolonged immobility, such as sitting in one position, is a risk factor for PIs, placing individuals with reduced mobility—such as wheelchair users with SCI—at a high risk of developing PIs [8]. In persons with SCI, prolonged pressure (e.g., on the buttocks) does not trigger feedback to elicit movement, unlike in persons without sensory or motor deficits. This lack of feedback can potentially lead to skin breakdown [8,9]. Other factors, such as completeness of lesion, independence in activities of daily living, or weight, can further influence the risk of developing PIs [10,11]. SP is likely the result of a disbalance between shoulder capacity and load caused by repetitive or strenuous tasks such as wheelchair propulsion, transfers, or reaching from a seated position [12]. Although the episodic onset of PIs is distinct from the chronic nature of SP, both health problems have a substantial impact on the individual and result in notable direct and indirect healthcare and societal costs [13,14,15,16,17].

Performing intermittent pressure relief (PR) is considered as one of the most effective strategies for preventing PI acquired from sitting [9,18]. PR is a movement that shifts the body weight off the buttocks, most commonly achieved by leaning forward or sidewards or pushing off the buttocks from the wheelchair cushion using both arms (i.e., PR lift) [19,20]. All three PR techniques have been shown to be effective and cost efficient for increasing oxygenation and blood flow in the buttocks [18,21]. However, the PR lift is also considered a risk factor for SP, as it places an additional load on the shoulders, which are already heavily loaded from activities of daily living [22,23,24]. Despite a shift in clinical recommendations towards adopting the PR techniques forward and side lean [25,26,27], the majority of wheelchair users still use the PR lift [19]. It also remains to be shown whether the forward and side lean are less likely to cause SP and whether they are as effective in preventing PIs as a PR lift. Clinical guidelines for PR recommend implementing pressure-relieving manoeuvres ranging from 15 s every 15 min to 60 s every hour. Based on self-report assessments and short-term measurements of approximately one week, the performance of PR (“PR performance”) in wheelchair users typically does not meet these guidelines [19,28]. Since self-reported assessments of PR performance may not be reliable [29], studies using objective measures to assess PR performance are needed. Furthermore, the long-term effectiveness of PR in preventing PI remains unclear, which highlights the need for long-term measurements.

To align and improve the current recommendations regarding PI and SP prevention, we present the protocol for our study to investigate the impact of PR performance on the occurrence of PI and SP in manual wheelchair users with SCI. The aim of this study is to contribute to an in-depth understanding of PR performance in the daily lives of wheelchair users with SCI and its complex association with the occurrence of PI and SP. The use of novel textile measurement mats [30] will facilitate the objective, individual quantification of PR performance in daily life several times over the course of one year. To account for the multifactorial nature of PI development, we will comprehensively assess a broad range of potential confounders, including physiological, psychological, and lifestyle factors (Appendix A, Table A1). We hypothesise that a higher relative PR time is related to a lower incidence of PI independent of the PR technique. Furthermore, we hypothesise that between-person differences in PR technique, but not in relative PR time, are related to the prevalence of SP.

## 2. Materials and Methods

### 2.1. Study Design and Participants

**Figure 1:**
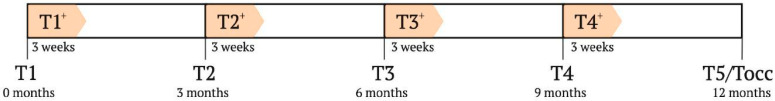
Study timeline. T1 to T5/Tocc = timepoints for assessment of confounders, three months apart; T1^+^ to T4^+^ = measurement periods of three weeks (continuous assessment of pressure relief performance and weekly assessment of shoulder pain) directly after T1 to T4.

This longitudinal observational study investigates the impact of PR performance on the occurrence of PI and SP in manual wheelchair users with SCI (Figure 1). The study protocol was designed according to the Standard Protocol Items: Recommendations for Interventional Trials 2013 Statement [31] (Supplement S1). The study is planned to run from March 2024 (first study visit) to March 2026 (last study visit). Study visits will take place either at the study centre (Swiss Paraplegic Research, Nottwil, Switzerland) or at the participant’s place of residence. Participants will receive compensation for travel and time expenses. This project is a nested project of the population-based Swiss Spinal Cord Injury cohort study (SwiSCI) [32].

Recruitment for this study is primarily carried out by screening the SwiSCI database for eligible participants. Individuals fulfilling the inclusion criteria according to the database are informed by the SwiSCI study centre about this study and invited to participate. Additional participants will be recruited through healthcare professionals and outpatient clinics at the four specialised SCI centres in Switzerland. Finally, information and calls for participation will be disseminated through magazines and social media channels of the SCI community. Individuals who express interest in participating in the study will be scheduled for a screening visit, which will be conducted either via phone or in person. During this visit, the inclusion and exclusion criteria (Table 1) will be reviewed to assess eligibility. Written informed consent (Supplement S2) is obtained from all participants prior to study initiation by qualified study employees.

We estimated the minimal sample size needed to detect the effect of PR performance (exposure of interest) on PI occurrence over time (outcome of interest). The hazard ratio was defined as the measure of effect [33]. Using the power calculation platform for proportional hazards regression (here, a Cox model) in Stata Version 18.0, the minimal sample size was calculated across a range of plausible hazard ratios for the event of a PI related to differences in PR performance (delta), while employing a plausible range of population-average probabilities for the event of a PI over the one-year study period.

**Figure 2:**
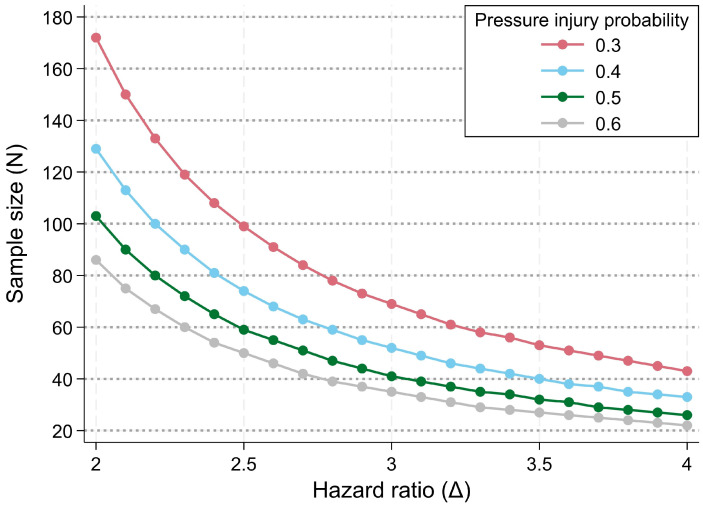
Calculation of the minimal sample size using Cox proportional hazards modelling. The x-axis indicates the hazard ratio (Δ) for the event of a pressure injury owing to differences in pressure relief performance. Presuming a population-average, one-year probability for pressure injury (event of interest) of 0.3, and conventionally accepting a power (1 − β) of 0.8 and a significance level (α) of 0.05, a minimal sample size of 60 participants is needed to detect an empirically plausible hazard ratio of 3.2 or higher.

Combining data on PI incidence from the SwiSCI community survey conducted in 2017 [34] with evidence from previous studies [35,36,37,38] on the comparative incidence of sitting-related PI’s, we obtained a minimal one-year event probability of a newly acquired sitting-related PI of approximately 0.3 to 0.4 for the study population. Using a conservative event probability of 0.3 and presuming a power (1 − β) of 0.8 as well as a significance level α of 0.05, the power calculation indicated that an achievable sample size of 60 participants supports the detection of hazard ratios of approximately 3.1 or higher (Figure 2).

Hazard ratios of such magnitude are plausible in SCI, as shown by Verschueren et al. [38], who analysed predictors of PI and reported adjusted odds ratios of 5.0 for the lesion level (tetraplegia vs. paraplegia). PR performance is seen as an important mediator from lesion level to PI development, since the ability to perform PR regularly decreases with higher lesion level.

A preliminary screening of the SwiSCI database in February 2024 identified 373 eligible individuals. We expected that approximately 75% would be eligible for participation. If 20% were willing to participate, we would have approximately 60 participants. With the additional recruitment within specialised SCI clinics, the recruitment of 70 participants is feasible.

### 2.2. Study Procedures and Outcomes

**Figure 3:**
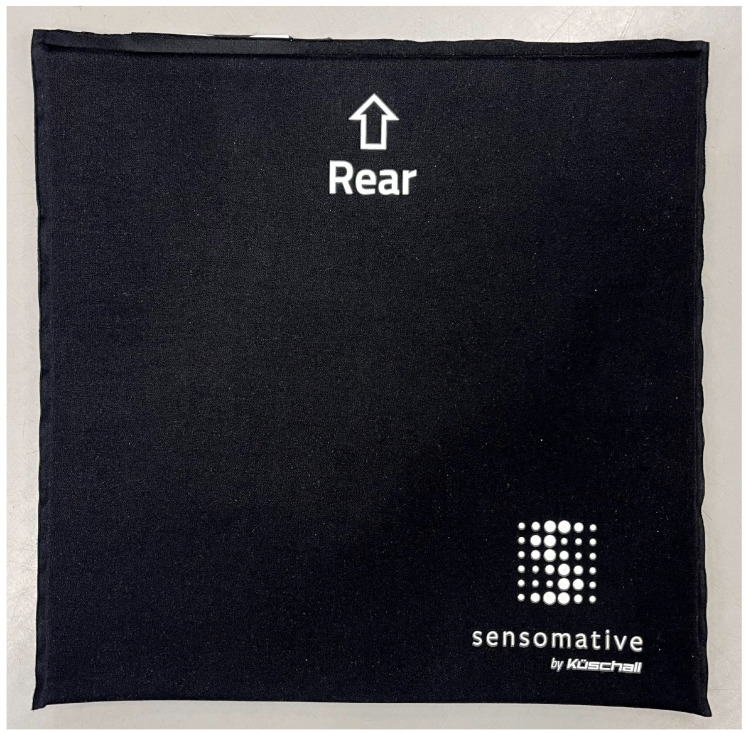
Pressure measurement mat.

Study participation includes five visits over the course of one year (Figure 1). Trained study employees perform all assessments according to a standardised protocol. An overview of the study procedures, including the time points at which each outcome and confounder will be assessed, is provided in the Appendix A (Table A1). The initial visit (T1) takes place at the study centre. Individual characteristics and potential confounding factors for PI and SP are collected using interviews, questionnaires, and non-invasive assessments. Potential confounders were identified based on the literature or expert opinion. Considering the multifactorial nature of PI and SP, including a wide range of confounding variables allows the estimation of each participant’s individual risk for PI and SP beyond their PR behaviour. PI occurrence will be self-reported and classified according to the National Pressure Injury Advisory Panel (NPIAP) staging system [7]. After T1, participants are assessed every three months (T2 to T5) either at the study centre or at their homes, depending on their personal preference. At each visit, the confounders for PI and SP are reassessed. At the end of each of the first four visits (T1 to T4), a textile measurement mat (Sensomative wheelchair, Sensomative GmbH, Rothenburg, Switzerland; Figure 3) is placed underneath the wheelchair cushions to quantify PR performance during the following three weeks (T1^+^ to T4^+^). The measurement mat includes 12 piezo-resistive textile sensors, each with a size of 4 cm × 4 cm which are distributed over the area of the mat (31 cm × 31 cm). Each sensor is continuously measuring pressure with a frequency of 5 Hz (pressure range: 0–60 kPa, resolution: 8 bits). The data of all sensors are transmitted to a connected data storage device using Bluetooth Low Energy. Following the method previously described by Hubli et al. [30], a calibration of the different PR techniques (PR lift, side lean, forward lean, and individual technique) is performed before and after each measurement, which allows for the classification of individual data. To ensure its position during the entire measurement period, the mat is fixed to the wheelchair cushion using tape or Velcro, depending on the type of the cushion.

**Figure 4:**
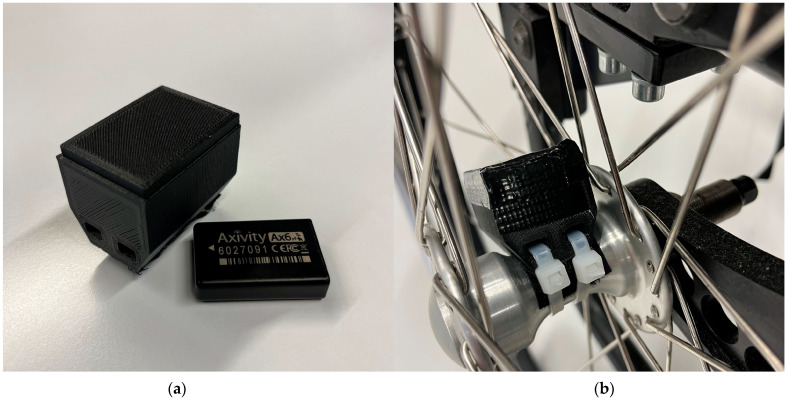
(**a**) Inertial measurement unit and plastic case; (**b**) installed setup.

Furthermore, an inertial measurement unit (IMU; Axivity AX6, Axivity Ltd., Newcastle upon Tyne, UK) sensor is attached to the wheel (Figure 4) to measure wheelchair mobility metrics as described by De Vries et al. [39] (i.e., distance covered, linear velocity of the wheelchair, number and duration of pushes, and number and magnitude of turns; measurement frequency: 100 Hz). Shoulder pain is assessed weekly at the end of each measurement week using the Wheelchair User’s Shoulder Pain Index (WUSPI) [40]. The WUSPI provides a validated tool for measuring shoulder pain intensity during functional activities of daily living in wheelchair users. This measure was selected to capture general activity-related shoulder pain that reflects the cumulative impact of daily shoulder loading, of which PR lifts are a significant contributor [22]. Furthermore, specific WUSPI items, such as transfers, may help relate shoulder load during PR lifts to SP. To ensure compliance with completing the WUSPI, participants will receive weekly reminders through their preferred communication channel (e.g., email or phone). If a PI occurs during the study, a final visit (Tocc) takes place according to the regular schedule and no further measurements or visits are conducted afterwards.

In the event of a sensor malfunction, data loss, or insufficient data quality, the respective measurement period will be repeated whenever feasible within the study timeline. If repeating a measurement is not possible, incomplete data from the affected measurement period will be excluded from analysis. Sensitivity analyses will be performed to assess potential bias resulting from missing data.

A comprehensive list of all study parameters and their corresponding assessments is provided in Appendix A (Table A1). At T1, individual characteristics and all confounders are assessed using interviews, questionnaires, or non-invasive assessments. PI occurrence, severity, and location are assessed at each visit. Parameters with high variability over time are also assessed from T2 to T5/Tocc. Other confounders are only assessed if the participants report changes since the previous visit. During the three-week measurement periods (T1^+^ to T4^+^), PR performance (including PR technique, PR frequency [times per seated hour], and PR duration [s]) is assessed continuously using the Sensomative measurement mat, and wheelchair mobility is measured with an IMU sensor. During the measurement periods, SP is assessed weekly using the WUSPI, a reliable and validated tool measuring shoulder pain intensity during functional activities of daily living in wheelchair users [40].

Researchers as well as clinicians with experience in the field of SCI were involved in the design of the study protocol. The study procedures were discussed and extensively tested with individuals with SCI. During these pilot tests, feedback was provided on the procedures, wearability, and the potential burden of study participation. It was reported that the sensors were not noticed during daily activities, nor did they lead to behavioural changes. Compliance with sensor use was high. To further reduce participant burden and increase adherence, study visits can also take place at the participant’s home.

This publication is based on version 2.0 of the study protocol, dated 19 February 2024. Recruitment began on 13 March 2024, and data collection is estimated to end in July 2026.

### 2.3. Quality Assurance and Safety Provisions

Data capture and management are performed using the web-based system SecuTrial (Interactive Systems, Berlin, Germany), which fulfils Good Clinical Practice requirements. Within the database, which is managed by a study-independent data manager, each study employee has predefined rights according to their role in the study. Errors during data entry are minimised by built-in control mechanisms and plausibility rules. The sensor data are stored on a secure server that only authorised study personnel have access to. Each participant is assigned a unique identification code. Documents allowing the identification of participants are stored separately on a secure server.

In addition to the safety measures concerning data management, the Swiss Paraplegic Research Clinical Trial Unit is assigned to perform study monitoring according to the study-specific monitoring plan. The monitoring plan is based on the risk-adapted monitoring approach described in the ADAMON study [41]. All source data and documents are accessible to the monitors. A monitoring report is made after every monitoring visit.

All assessments and study procedures are non-invasive and are expected to pose no excessive risks or burden to the participants. Quality measures are in place to minimise the risk of unauthorised data access or the unintentional identification of participants. Serious events are documented and reported to the Sponsor-Investigator and the responsible Ethics Committee during the entire study period.

Participants are allowed to withdraw from the study at any time without providing a reason. Participants are excluded from the study if the eligibility criteria are no longer fulfilled. In the event of exclusion or withdrawal of consent, all data collected up to this point are used in coded form for the analysis.

### 2.4. Data Analysis Plan

Processing of the data from the pressure measurement mat will be carried out as described by Hubli et al. [30]. During the individual calibration before and after the measurement period, the system is customised to account for individual characteristics by assessing the user’s different PR techniques and an upright reference sitting position. After the measurement period, the collected data—comprising twelve pressure values per measurement frame—are classified using a random forest supervised learning algorithm using the calibration data. The algorithm distinguishes between the regular sitting position and various PR positions. This classification approach was previously developed by Zemp et al. and has been validated and applied in several previous studies [30,42,43]. Furthermore, the pilot testing we conducted prior to the start of our study yielded good results in terms of accuracy, precision, and sensitivity. The daily duration of sitting time and the used PR techniques as well as the according duration and frequency will be analysed and described descriptively.

Statistical data analysis will be performed using the contemporary versions of the statistical software packages STATA (Version 18.0, StataCorp LLC, College Station, TX, USA), R (Version 4.5.0, GNU General Public License, Free Software Foundation, Boston, MA, USA), RStudio (Version 2025.05, Posit PBC, Boston, MA, USA), Matlab (Version R2025a, MathWorks, Natick, MA, USA), and Python (Version 3.13.4, Python Software Foundation, Wilmington, DE, USA). The data analysis plan includes the use of basic descriptive statistics, as well as univariable and multivariable regression models that are appropriate for the error distribution of the outcome at stake. Descriptive and univariable statistics will be used to describe the study population and the outcomes at the different measurement points. Univariable analyses of between-group differences will make use of basic statistics, including chi-squared or Fisher’s exact tests for categorical variables and t-tests or non-parametric Kruskal–Wallis tests for continuous variables. Descriptive analysis of time-to-event outcomes (PI occurrence) will include Kaplan–Meier and Nelson–Aalen cumulative hazard function plots. The assessment and updating of a wide range of potentially confounding parameters (Appendix A, Table A1) allows for statistical control of within-person and between-person variation in the primary outcomes and helps to understand the complex association of PR performance with PI and SP. Furthermore, support for a causal association of PR performance with PI and SP will be inferred using directed acyclic graphs and multivariable regression modelling. To account for missing data in multivariate analysis, we will use the method of multiple imputation using chained equations and assuming missing data at random [44]. To assess the robustness of the findings, pattern mixture sensitivity analysis will be considered for scenarios indicating substantial missingness (>10%) of a non-random pattern nature [45].

No interim analyses are planned for this study. In case of a serious event, the study will be stopped until a safety review is completed. In such cases, interim analyses will be performed.

### 2.5. Dissemination Policy

The results of this study will be published in peer-reviewed journals and presented at scientific conferences. Results will also be disseminated through newsletters and the online media of the Swiss Paraplegic Association. Individual results will be shared with the participants at the end of the study. Anonymised data and statistical codes will be made available upon request.

## 3. Strengths and Limitations

One of the key strengths of this study is that it is the first to objectively assess pressure relief performance in the daily lives of participants with SCI over an extended period of time. The longitudinal design with multiple measurement time points offers valuable insight into PR performance in daily life. Compared to previous studies, which measured PR performance over shorter periods or relied on self-reports, our study design enables us to provide more reliable information on PR performance in daily life. Furthermore, it allows us to infer causality in the complex relationship between PR performance, PI, and SP. The evaluation of a wide range of relevant confounders, which were selected based on the literature and expert opinion, allows us to investigate the relationship even more comprehensively. We acknowledge that achieving the target sample size of 70 participants may be challenging, given the limited size of the target population of individuals with complete SCI living in Switzerland per se as well as the relatively long duration of participation for the participants. Nevertheless, we are confident that we will be able to achieve this sample size using the different recruitment strategies presented.

## 4. Outlook

The results of this study provide valuable insight into the complex relationship between PR performance, PI, and SP. This can support clinical decision making and improve clinical guidelines for PR. Furthermore, this study will establish a foundation for future interventional research on PR performance. Such studies may involve the comparison of different PR strategies with respect to their effectiveness in preventing PI and/or SP. The ultimate objective is to reduce the incidence of both PI and SP in community-dwelling individuals with SCI.

## Figures and Tables

**Table 1:** Eligibility criteria assessed during the screening visit.

Inclusion Criteria	Exclusion Criteria
Persons with a complete spinal cord injury (AIS A) Age ≥ 18 years Time since injury ≥ 5 years Main mode of mobility: manual wheelchair Score of 1 or 2 in the SCIM-SR item “Mobility for Moderate Distances”: needs electric wheelchair or partial assistance to operate manual wheelchair (1) or moves independently in manual wheelchair (2) Ability to perform pressure relief independently or with the aid of the wheelchair or a table Living in Switzerland	Total daily sitting time < 8 h Need of a specialised assistive device for performing pressure relief Current hospitalisation Current pressure injury Pregnancy (anamnestic)

Note: AIS = American Spinal Injury Association Impairment Scale, SCIM-SR = Spinal Cord Independence Measure Self-Report.

## Data Availability

No new data were created or analysed in this study. Data sharing is not applicable to this article.

## Supplementary Materials

The following supporting information can be downloaded at: https://www.mdpi.com/article/10.3390/mps8030062/s1, Supplement S1: SPIRIT Checklist for the study protocol of the PRperf study; Supplement S2: Participant information and informed consent form.

## Abbreviations

The following abbreviations are used in this manuscript:

AIS	American Spinal Injury Association Impairment Scale
EKNZ	Swiss Ethics Committee for Northwest/Central Switzerland
IMU	Inertial measurement unit
PI	Pressure injury
PR	Pressure relief
PRperf	Pressure relief performance
SCI	Spinal cord injury
SCIM-SR	Spinal Cord Independence Measure Self-Report
SP	Shoulder pain
SwiSCI	Swiss Spinal Cord Injury cohort study
WUSPI	Wheelchair User’s Shoulder Pain Index

## Appendix A

**Table A1:** Detailed assessments table of primary outcomes and confounders.

Exposure and Main Outcomes	Assessment Method	Parameter/Unit	Assessment Timepoints	Literature	
PR performance	Measured using a textile sensor mat (Sensomative wheelchair, Sensomative GmbH, Rothenburg, Switzerland)	PR technique (weight relief lift, forward lean, side lean, or other) PR frequency (times/h) PR duration (s)	**T1^+^, T2^+^, T3^+^, T4^+^**	[30]	
PI occurrence	Questioning	Location and grade (following National Pressure Injury Advisory Panel (NPIAP) pressure injury stages 1 to 4, including unstageable pressure injuries)	**Screening, T1, T2, T3, T4, T5/Tocc**	[7,46]	
SP	Wheelchair User’s Shoulder Pain Index (WUSPI)	Score of 0 to 150 points	**T1^+^, T2^+^, T3^+^, T4^+^**	[40]	
**Confounder**	**Assessment Method**	**Parameter/Unit**	**Assessment Timepoints**	**Confounder for**	**Literature**
Age	Questioning	Years	Screening	PI, SP	[11,12,36,47,48,49,50]
Alcohol consumption	Questioning	Days/week and units/day	**T1, T2, T3, T4, T5/Tocc**	PI	[51]
Anxiety and depression	Hospital Anxiety and Depression Scale (HADS)	Score of 0 to 21 points for anxiety and depression, respectively	**T1, T2, T3, T4, T5/Tocc**	PI, SP	[48,52]
Autonomic dysreflexia	ISAFSCI questionnaire	Binary (yes/no)	**T1, T2, T3, T4, T5/Tocc**	PI	[10,53]
Bed rest during daytime/day	Questioning	Hours	**T1^+^, T2^+^, T3^+^, T4^+^**	PI	
Diabetes mellitus	Questioning	Binary (yes/no)	**T1, (T2), (T3), (T4), (T5/Tocc)**	PI	[50,54,55]
Feverish infection	Questioning	Binary (yes/no)	**T1, T2, T3, T4, T5/Tocc**	PI	[10,54]
Height	Questioning	cm	**T1**	PI, SP	
Incontinence	ISAFSCI questionnaire	Score of 0 to 2 points	**T1, T2, T3, T4, T5/Tocc**	PI	[11,48,53,54]
Independence in activities of daily life	SCIM-SR questionnaire	Score of 0 to 100 points; divisible into three subscales	**T1, (T2), (T3), (T4), (T5/Tocc)**	PI, SP	[10,54,55,56]
Lesion AIS	Questioning, Medical record	Score of A to E	**T1, (T2), (T3), (T4), (T5/Tocc)**	PI, SP	[2,10,12,36,49,55]
Lesion duration	Questioning, Medical record	Years since injury	**Screening**	PI, SP	[12,22,36,47,49,57]
Lesion level	Questioning, Medical record	C1 to S5	**T1, (T2), (T3), (T4), (T5/Tocc)**	PI, SP	[2,12,22,49,57]
Medication intake	Questioning	Type of medication	**T1, T2, T3, T4, T5/Tocc**	PI, SP	[47,57]
Menopausal status	Questioning	Binary (menopause yes/no)	**T1, T2, T3, T4, T5/Tocc**	PI	
Mobility independence	SCIM-SR questionnaire—Mobility during short distances (10–100 m)	Score of 0 to 8	**Screening, T1, T2, T3, T4, T5/Tocc**	PI, SP	[11,56]
MVPA duration/week	Questioning	Hours/week	**T1, T2, T3, T4, T5/Tocc**	PI, SP	
PI location	Questioning	Location	**T1, T2, T3, T4, T5/Tocc**	PI	[11,48,55]
PI stage	Questioning	Stage 1 to 4	**T1, T2, T3, T4, T5/Tocc**	PI	[7,11,46,48,54]
PI treatment	Questioning	Conservative/surgical	**T1, T2, T3, T4, T5/Tocc**	PI	
Pressure relief quality	Pressure mat (ForeSite SS, Xsensor, Calgary, Canada)	% of pressure compared to upright sitting	**T1, (T2), (T3), (T4), (T5/Tocc)**	PI	
RoM upper extremity	Goniometer	Degrees	**T1, (T2), (T3), (T4), (T5/Tocc)**	SP	[22]
Rotator cuff function	Belly-Press Test (also Napoleon-Test)	Binary (positive/negative)	**T1, (T2), (T3), (T4), (T5/Tocc)**	SP	[58,59]
Self-efficacy	General self-efficacy scale	Score from 0 to 40	**T1, T2, T3, T4, T5/Tocc**	PI, SP	[60,61,62]
Sex	Questioning	Binary (male/female)	**Screening**	PI, SP	[2,37,49]
Shoulder injuries	Questioning	Type of injury	**T1, (T2), (T3), (T4), (T5/Tocc)**	SP	[12]
Shoulder intervention	Questioning	Type of intervention	**T1, (T2), (T3), (T4), (T5/Tocc)**	SP	
Shoulder joint disease/pathology	Questioning	Type of disease/pathology	**T1, (T2), (T3), (T4), (T5/Tocc)**	SP	[12,22,58]
Sitting time/day	Questioning during screening, afterwards using measurement mat (Sensomative)	Hours	**Screening, T1^+^, T2^+^, T3^+^, T4^+^**	PI, SP	
Skin check performance	Questioning	Times/week	**T1, T2, T3, T4, T5/Tocc**	PI	[11]
Smoking	Questioning	Cigarettes/day	**T1, T2, T3, T4, T5/Tocc**	PI	[47,51,55,57]
Spasticity lower extremities	Modified Ashworth Scale	Score from 0 to 4	**T1, (T2), (T3), (T4), (T5/Tocc)**	PI	[2,48,63]
Spasticity upper extremities	Modified Ashworth Scale	Score from 0 to 4	**T1, (T2), (T3), (T4), (T5/Tocc)**	SP	[2,48,63]
SR-PR performance	Questioning	Times/hour and seconds/PR	**T1^+^, T2^+^, T3^+^, T4^+^**	PI	
Transfer number/day	Measurement mat (Sensomative)	Times/day	**T1^+^, T2^+^, T3^+^, T4^+^**	PI, SP	[11,64]
Transfer quality	Transfer Assessment Instrument	Score from 0 to 10	**T1, (T2), (T3), (T4), (T5/Tocc)**	PI, SP	[11,64,65,66]
Weight	Measurement on a wheelchair-accessible scale, measurement of wheelchair separately from participant	kg	**T1, (T2), (T3), (T4), (T5/Tocc)**	PI, SP	[11,12,22,48,50,55]
Wheelchair hip angle	Goniometer	Degrees	**T1, (T2), (T3), (T4), (T5/Tocc)**	PI	[12]
Wheelchair shoulder position	Assessment	In front of hip/above hip/behind hip	**T1, (T2), (T3), (T4), (T5/Tocc)**	SP	[12]
Wheelchair cushion type	Questioning	Cushion brand and model	**T1, (T2), (T3), (T4), (T5/Tocc)**	PI	[11,67]
Wheelchair mobility	Wearable IMU sensor attached to wheelchair wheel (Axivity AX6, Axivity Ltd., Newcastle upon Tyne, UK)	Distance covered (km/day), linear velocity of the wheelchair (km/h), number of pushes (pushes/day), duration of pushes (s/push), number of turns (turns/day), magnitude of turns (degrees/turn)	**T1^+^, T2^+^, T3^+^, T4^+^**	PI, SP	[22,39]

Note. AIS = American Spinal Cord Injury (AISA) Impairment Scale; IMU = Inertial Measurement Unit; ISAFSCI = International Standards to Document Remaining Autonomic Function After Spinal Cord Injury; MVPA = Moderate-to-Vigorous Intensity Physical Activity; PI = pressure injuries; PR performance = pressure relief performance; RoM = range of motion; SP = shoulder pain; T1, T2, T3, T4, T5 = study visits; T1^+^, T2^+^, T3^+^, T4^+^ = measurement periods of three weeks; (T2), (T3), (T4), (T5/Tocc) = update, assessment only performed if necessary (changes since baseline measurement).
