# Supplementary material for: Investigating the Impact of Pressure Relief Performance on the Occurrence of Pressure Injuries and Shoulder Pain in Wheelchair Users with Spinal Cord Injury (PRperf Study): Study Protocol for a Prospective Observational Study
Source: Methods Protoc. 2025 Jun 6;8(3):62. doi: 10.3390/mps8030062 (PMC12196044; doi:10.3390/mps8030062)
Supplement: Supplementary file 1 [file mps-08-00062-s001.zip › mps-3610503-supply/Supplement_2_ICF.pdf]

Supplement 2: Participant information and informed consent form

Anfrage zur Teilnahme an klinischer Forschung:

Schweizer Paraplegiker Forschung

Druckentlastung und das Auftreten von Dekubitus und Schulterschmerzen: Eine Beobachtungsstudie bei Rollstuhlfahrer*innen mit Querschnittlähmung

Sehr geehrte Dame, sehr geehrter Herr

Wir fragen Sie hier an, ob Sie bereit wären, an unserem Forschungsvorhaben mitzuwirken.
Ihre Teilnahme ist freiwillig. Alle Daten, die in diesem Forschungsprojekt erhoben werden, unterliegen strengen Datenschutzvorschriften.
Das Forschungsvorhaben wird von der Schweizer Paraplegiker Forschung durchgeführt. Bei Interesse informieren wir Sie gerne über die Ergebnisse aus diesem Forschungsprojekt.
In einem Gespräch erklären wir Ihnen die wichtigsten Punkte und beantworten Ihre Fragen. Damit Sie sich bereits jetzt ein Bild machen können, hier das Wichtigste vorweg. Im Anschluss folgen dann weitere, detailliertere Informationen.

Warum führen wir dieses Forschungsvorhaben durch?

- Dekubitus (Wundliegen/Druckgeschwüre) und Schulterschmerzen sind bei Personen mit einer Querschnittlähmung weit verbreitet. Die Technik, Häufigkeit und Dauer der Druckentlastung könnten einen Einfluss auf das Auftreten der genannten Probleme haben.
- In dieser Studie möchten wir den Zusammenhang zwischen Druckentlastungsverhalten und Dekubitus und Schulterschmerzen untersuchen.

Was muss ich bei einer Teilnahme tun? – Was geschieht mit mir bei einer Teilnahme?

- Form der Teilnahme: Wenn Sie sich entscheiden mitzumachen, müssen Sie einige Fragen beantworten, Fragenbögen ausfüllen, und mit Ihnen werden einige Untersuchungen durchgeführt.
- Ablauf der Teilnahme: Wenn Sie teilnehmen, führen wir über ein Jahr verteilt fünf Studienbesuche durch, nach Ihrer persönlichen Präferenz entweder in Nottwil oder bei Ihnen zuhause. Bei den ersten vier Studienbesuchen wird eine textile Messmatte unter Ihr Rollstuhlkissen gelegt, welche in den darauffolgenden drei Wochen Ihr Druckentlastungsverhalten misst.

Welcher Nutzen und welche Risiken sind mit einer Studienteilnahme verbunden?

Nutzen

- Mit Ihrer Teilnahme an dieser Studie helfen Sie uns, den Zusammenhang von Druckentlastungen mit Dekubitus und Schulterbeschwerden besser zu verstehen.
- Daraus können wir Empfehlungen bezüglich Druckentlastungsverhalten ableiten, von denen später Sie selbst und andere von Querschnittlähmung betroffene Personen profitieren können.
- Nach Abschluss der Studie erhalten Sie von uns eine Auswertung Ihrer persönlichen Ergebnisse.

Risiken und Belastung

- Die Teilnahme an der Studie ist mit sehr minimalen Risiken verbunden.
- Die Gesamtstudiendauer von einem Jahr, sowie die wiederholten Studienbesuche können eine Belastung darstellen. Aus diesem Grund besteht die Möglichkeit, die meisten Studienbesuche bei Ihnen zuhause durchzuführen.
- Es gibt Sicherheitsmassnahmen, die das Risiko eines unbefugten Datenzugriffs und/oder einer ungewollten Identifizierung von Teilnehmer*innen auf ein Minimum reduzieren.

Mit Ihrer Unterschrift am Ende des Dokuments bezeugen Sie, dass Sie freiwillig teilnehmen und dass Sie die Inhalte des gesamten Dokuments verstanden haben.

Detaillierte Information

1. Ziel und Auswahl

Unser Forschungsvorhaben bezeichnen wir in dieser Informationsschrift als *Forschungsprojekt.* Wenn Sie an diesem Forschungsprojekt teilnehmen, sind Sie *ein*e Teilnehmer*in*.

In diesem Forschungsprojekt wollen wir untersuchen, wie das Druckentlastungsverhalten im Alltag und das Auftreten von Dekubitus und Schulterschmerzen bei querschnittgelähmten Rollstuhlfahrer*innen zusammenhängt. Wir fragen Sie an, da alle Personen teilnehmen können, die:

- Mindestens 18 Jahre alt sind
- In der Schweiz leben
- Seit mindestens fünf Jahren eine Querschnittlähmung haben
- Vollständig gelähmt sind
- Bei ihren täglichen Aktivitäten auf einen Rollstuhl angewiesen sind
- Druckentlastungen selbstständig und ohne Hilfsmittel (z.B. Kippfunktion im Elektrorollstuhl) durchführen können
- Täglich mindestens acht Stunden sitzen
- Zurzeit nicht hospitalisiert sind
- Zurzeit keinen Dekubitus aufweisen
- Zurzeit nicht schwanger sind

2. Allgemeine Informationen

Dekubitus und Schulterschmerzen sind zwei weit verbreitete und folgenschwere Gesundheitsprobleme bei Menschen mit Querschnittlähmung, die auf einen Rollstuhl angewiesen sind. Regelmässige Druckentlastung wird als eine der besten Strategien zur Vorbeugung von Dekubitus angesehen, gilt aber gleichzeitig je nach Ausführung als Risikofaktor für Schulterschmerzen. Mit diesem Forschungsprojekt möchten wir den Zusammenhang zwischen dem Druckentlastungsverhalten im Alltag und dem Auftreten von Schulterschmerzen und Dekubitus bei querschnittgelähmten Rollstuhlfahrer*innen untersuchen. Wenn Sie am Forschungsprojekt teilnehmen, werden wir fünfmal innerhalb eines Jahres einige Untersuchungen mit Ihnen durchführen. Ausserdem müssen Sie einige Fragen beantworten und Fragebögen ausfüllen. Der Zeitaufwand beträgt etwa 1.5-2 Stunden pro Studienvisite (erste Visite etwas länger, ca. 3-4 Stunden). Bei den ersten vier Studienvisiten legen wir eine textile Messmatte unter Ihr Rollstuhlkissen und messen während drei Wochen Ihr Druckentlastungsverhalten. Das Forschungsprojekt wird von der Schweizer Paraplegiker Forschung durchgeführt. Das Ziel ist, 70 Studienteilnehmer*innen einzuschliessen.
Die Durchführung der Studie entspricht allen gesetzlichen Vorgaben der Schweiz. Ausserdem beachten wir alle international anerkannten Richtlinien. Die zuständige Ethikkommission hat das Forschungsprojekt geprüft und bewilligt.

3. Ablauf

Zu Beginn werden wir überprüfen, ob Sie die Ein- und Ausschlusskriterien erfüllen. Falls Sie alle Kriterien erfüllen und sich zu einer Studienteilnahme entscheiden, werden wir Ihnen die Einverständniserklärung zustellen, welche sowohl von Ihnen als auch von uns unterschrieben werden muss, damit die Studienteilnahme beginnen kann.

Im Abstand von jeweils drei Monaten werden wir über ein Jahr hinweg einige mit Dekubitus und Schulterbeschwerden in Zusammenhang stehende Untersuchungen durchführen (siehe Abbildung 1; T1 bis T5/Tocc). Ausserdem werden Sie an diesen insgesamt fünf Terminen einige Fragen beantworten und Fragebögen ausfüllen. Diese dienen dazu, uns ein ganzheitliches Bild der mit Dekubitus und Schulterbeschwerden in Zusammenhang stehenden Einflussfaktoren zu geben. Die erste Visite ist ausführlicher (ca. 3-4 Stunden) und wird in Nottwil an der Schweizer Paraplegiker Forschung durchgeführt. Die restlichen vier Studienvisiten dauern ca. 1.5-2 Stunden und werden zu einem Zeitpunkt und an einem Ort durchgeführt, der Ihnen am besten passt (z.B. bei uns in Nottwil oder bei Ihnen zuhause). Bei den ersten vier Visiten werden wir jeweils eine textile Messmatte unter Ihr Rollstuhlkissen legen und während den darauffolgenden drei Wochen Ihr Druckentlastungsverhalten messen (siehe Abbildung 1; T1+ bis T4+). Während dieser Wochen können Sie Ihrem normalen Tagesablauf nachgehen, ohne von der Messmatte eingeschränkt zu werden, und einmal pro Woche müssen Sie einen Fragebogen ausfüllen, der uns zu Ihren Schulterbeschwerden Auskunft gibt (ca. 10 Minuten). Trotz vorgängiger Abklärung der Ein- und Ausschlusskriterien kann es sein, dass wir Sie im Laufe des Forschungsprojekts ausschliessen müssen, falls Sie diese nicht mehr erfüllen sollten.

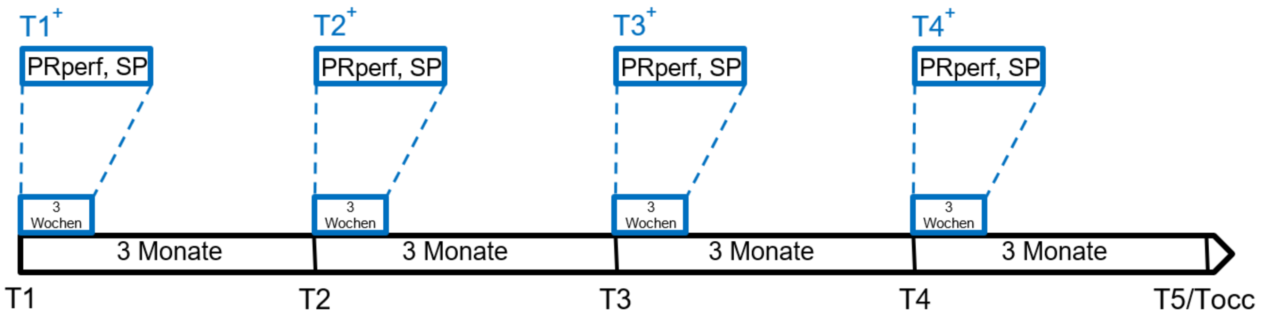

Abbildung 1: Projektablauf. T1 bis T5/Tocc: Studienvisiten (T1 ca. 3-4 h, T2-T5/Tocc ca. 1.5-2 h); T1+ bis T4+: Messungen (Fragebogen ca. 10 Minuten pro Woche); PRperf = Druckentlastungsverhalten; SP = Schulterschmerzen.

4. Nutzen

Mit Ihrer Teilnahme an dieser Studie leisten Sie einen wichtigen Beitrag zur Forschung im Bereich Dekubitus und Schultergesundheit. Sie helfen uns, den Zusammenhang von Druckentlastungen mit Dekubitus und Schulterbeschwerden besser zu verstehen. Daraus können wir Empfehlungen bezüglich Druckentlastungsverhalten ableiten, von denen später Sie selbst und andere von Querschnittlähmung betroffene Personen profitieren können. Sofern gewünscht, erhalten Sie nach Abschluss der Studie ausserdem eine Auswertung Ihrer persönlichen Studienergebnisse.

5. Freiwilligkeit und Pflichten

Sie nehmen freiwillig teil. Wenn Sie nicht an diesem Forschungsprojekt teilnehmen oder später Ihre Teilnahme zurückziehen wollen, müssen Sie dies nicht begründen. Ihre Behandlung/Betreuung ist unabhängig von Ihrem Entscheid gewährleistet.

Wenn Sie an diesem Forschungsprojekt teilnehmen, werden Sie gebeten:

- Sich an die Vorgaben und Anforderungen des Forschungsprojekts durch den Prüfplan zu halten (z.B. die Messmatte während der gesamten Messperiode im Rollstuhl lassen)
- Die Fragebögen ehrlich und wahrheitsgetreu auszufüllen
- Die Projektleitung zu informieren, wenn ein Dekubitus entsteht oder andere Beschwerden auftreten, welche eine weitere Teilnahme verhindern
- Die Projektleitung zu informieren, wenn eine Schwangerschaft vorliegt

6. Risiken und Belastungen

Durch das Forschungsprojekt sind Sie nur sehr geringfügigen Risiken ausgesetzt, da nur nicht-invasive und risikoarme Untersuchungen sowie Befragungen vorgesehen sind. Die Mehrheit der Untersuchungen wird routinemässig in der Klinik genutzt. Es sind Qualitätsmaßnahmen vorhanden, um das Risiko eines unbefugten Datenzugriffs und/oder einer unbeabsichtigten Identifizierung von Teilnehmer*innen zu minimieren.
Die lange Studiendauer sowie die lange Dauer der einzelnen Studienbesuche kann eine Belastung darstellen. Aus diesem Grund besteht die Möglichkeit, die meisten Studienbesuche bei Ihnen zuhause durchzuführen. Einige in der Studie eingesetzten Fragebögen beinhalten Fragen zur eigenen Persönlichkeit. Dies kann für manche Personen belastend sein. Hierbei wird auf das Recht aufmerksam gemacht, dass Sie die Studienteilnahme jederzeit und ohne Angabe von Gründen abbrechen dürfen.

Für Frauen, die schwanger werden können

Sollten Sie während des Forschungsprojekts schwanger werden, müssen Sie die Projektleitung informieren und dürfen nicht weiter an dem Forschungsprojekt teilnehmen. Der Projektleitung wird mit Ihnen das weitere Vorgehen besprechen.

7. Alternativen

Wenn Sie nicht an diesem Forschungsprojekt teilnehmen möchten, aber offen für andere Forschungsprojekte sind, sprechen Sie bitte mit der Projektleitung.

8. Ergebnisse

Es gibt:
1) *individuelle Ergebnisse des Forschungsprojekts*, die Sie direkt betreffen. Die Projektleitung wird Sie im Verlauf des Projekts über alle für Sie persönlich wichtigen neuen Ergebnisse und Erkenntnisse informieren. Sie werden mündlich und schriftlich informiert und können dann erneut entscheiden, ob Sie an dem Projekt weiter teilnehmen möchten.
2) *individuelle Ergebnisse des Forschungsprojekts, die zufällig entstehen (*sogenannte Zufallsergebnisse). Zufallsbefunde sind sogenannte „Begleit-Ergebnisse“, also Ergebnisse, nach denen man nicht explizit geforscht hat, sondern die zufällig gefunden werden. Bei Zufallsbefunden werden Sie informiert, wenn diese Befunde relevant für Ihre Gesundheit sind. Das bedeutet, dass solche Befunde Ihnen dann mitgeteilt werden, wenn man zufällig eine bislang nicht bekannte Erkrankung festgestellt hat oder eine noch nicht aufgetretene Erkrankung durch Vorbeugung verhindern kann. Wenn Sie darüber nicht informiert werden wollen, sprechen Sie bitte mit der Projektleitung
3) *objektive End-Ergebnisse des gesamten Forschungsprojekts*. Die Projektleitung kann Ihnen am Ende des Forschungsprojekts eine Zusammenfassung der Gesamtergebnisse zukommen lassen.

9. Vertraulichkeit von Daten und Proben

9.1. Datenverarbeitung und Verschlüsselung

Für dieses Forschungsprojekt werden Daten zu Ihrer Person und Gesundheit erfasst und bearbeitet, teilweise in automatisierter Form. Bei der Datenerhebung werden Ihre Daten verschlüsselt. Verschlüsselung bedeutet, dass alle Bezugsdaten, die Sie identifizieren könnten (Name, Geburtsdatum etc.), gelöscht und durch einen Code ersetzt werden. Personen, die keinen Zugang zu dieser Schlüssel-Liste haben, können keine Rückschlüsse auf Ihre Person ziehen. Die Schlüssel-Liste bleibt immer in der Schweizer Paraplegiker Forschung.
Nur sehr wenige Fachpersonen werden Ihre unverschlüsselten Daten sehen, und zwar nur, um Aufgaben im Rahmen des Forschungsprojekts zu erfüllen. Diese Personen unterliegen der Schweigepflicht. Sie als teilnehmende Person haben das Recht auf Einsicht in Ihre Daten.

9.2. Datenschutz

Alle Vorgaben des Datenschutzes werden streng eingehalten. Es ist möglich, dass Ihre Daten in verschlüsselter Form, zum Beispiel für eine Publikation, übermittelt werden müssen und anderen Forschern zur Verfügung gestellt werden können. Vor Ort gespeicherte gesundheitsbezogene Daten werden in einer Datenbank gesichert, die ausschließlich Forschungszwecken dient. Daten können verschlüsselt im Rahmen dieses Projekts in eine andere Datenbank versendet werden.

Die Projektleitung ist dafür verantwortlich zu sorgen, dass im Ausland die gleichen Standards wie in der Schweiz eingehalten werden.
Ärztinnen und Ärzte, die für die Nachbehandlung verantwortlich sind, können kontaktiert werden, um Auskunft über Ihren Gesundheitszustand zu geben.

9.3. Datenschutz bei Weiterverwendung

Ihre Daten könnten für die Beantwortung von anderen Fragestellungen zu einem späteren Zeitpunkt wichtig sein und/oder später an eine andere Datenbank der Schweiz oder ins Ausland für noch nicht näher definierte Untersuchungen (Weiterverwendung) versandt und verwendet werden. Diese andere Datenbank muss die gleichen Standards einhalten wie die Datenbank zu diesem Projekt.
Für diese Weiterverwendung bitten wir Sie, ganz am Ende dieses Dokuments eine weitere Einwilligungserklärung zu unterzeichnen. Diese zweite Einwilligung ist unabhängig von der Teilnahme an diesem Projekt.

9.4. Einsichtsrechte bei Kontrollen

Dieses Forschungsprojekt kann durch die zuständige Ethikkommission überprüft werden. Die Projektleitung muss dann Ihre Daten für solche Kontrollen offenlegen. Alle müssen absolute Vertraulichkeit wahren.

10. Rücktritt

Sie können jederzeit von dem Forschungsprojekt zurücktreten. Die bis dahin erhobenen Daten und Proben werden in diesem Fall allerdings noch verschlüsselt ausgewertet.
Im Falle eines Rücktritts bleiben Ihre Daten und Proben weiterhin verschlüsselt in den Projektdokumenten. Dies dient Ihrer medizinischen Sicherheit. Prüfen Sie bitte, ob Sie damit einverstanden sind, bevor Sie am Projekt teilnehmen.

11. Entschädigung

Wenn Sie bei diesem Forschungsprojekt mitmachen, erhalten Sie dafür folgende Entschädigung: CHF150 bei abgeschlossener Studienteilnahme. Falls die Studienteilnahme vorzeitig beendet wird, erhalten Sie CHF30 pro absolvierte Studienvisite. Es entstehen Ihnen oder Ihrer Krankenkasse keine Kosten durch die Teilnahme.
Die Ergebnisse dieses Forschungsprojekts können unter Umständen dazu beitragen, kommerzielle Produkte zu entwickeln. Durch Ihre Teilnahme haben Sie kein Anrecht auf Anspruch an kommerziellen Entwicklungen (z.B. Patente).

12. Haftung

Falls Sie durch das Forschungsprojekt einen Schaden erleiden sollten, haftet die Schweizer Paraplegiker-Forschung, die das Forschungsprojekt veranlasst hat und für die Durchführung verantwortlich ist. Die Voraussetzungen und das Vorgehen sind gesetzlich geregelt. Wenn Sie einen Schaden erlitten haben, so wenden Sie sich bitte an die Projektleitung.

13. Finanzierung

Das Forschungsprojekt wird vollständig von der Forschungskommission der Schweizer Paraplegiker Stiftung und der Schweizer Paraplegiker Forschung bezahlt.

14. Kontaktperson

Sie dürfen jederzeit Fragen zur Projektteilnahme stellen. Auch bei Unsicherheiten, die während des Forschungsprojekts oder danach auftreten, wenden Sie sich bitte an eine der unten aufgeführten Personen:

Yannik Schürch (Studienmitarbeiter):
Schweizer Paraplegiker-Forschung
Guido A. Zäch Strasse 4
6207 Nottwil
yannik.schuerch@paraplegie.ch
+41 41 939 65 93

Dr. Ursina Arnet (Studienleiterin):
Schweizer Paraplegiker-Forschung
Guido A. Zäch Strasse 4
6207 Nottwil
ursina.arnet@paraplegie.ch
+41 41 939 65 99

Einwilligungserklärung

Schriftliche Einwilligungserklärung zur Teilnahme an einem Forschungsprojekt
Bitte lesen Sie dieses Formular sorgfältig durch. Bitte fragen Sie, wenn Sie etwas nicht verstehen oder wissen möchten. Für die Teilnahme ist Ihre schriftliche Einwilligung notwendig.

**BASEC-Nummer:**	2024-00087
**Titel des Forschungsprojekts (wissenschaftlich und Laiensprache)**:	Druckentlastung und das Auftreten von Dekubitus und Schulterschmerzen: eine prospektive Beobachtungsstudie bei Rollstuhlfahrer*innen mit Querschnittlähmung (wissenschaftlich)<br><br>Zusammenhang von Druckentlastung, Dekubitus und Schulterschmerzen (Laien)
**Verantwortliche Institution (Projektleitung mit Adresse**):	Dr. Ursina Arnet<br>Schweizer Paraplegiker Forschung<br>Guido A. Zäch Strasse 4, 6207 Nottwil
**Ort der Durchführung**:	Schweizer Paraplegiker Forschung<br>Guido A. Zäch Strasse 4, 6207 Nottwil
**Studienleiter/in oder Studienmitarbeiter/in:**<br>Name und Vorname in Druckbuchstaben:	
**Teilnehmerin/Teilnehmer:**<br>Name und Vorname in Druckbuchstaben:<br><br>Geburtsdatum:	

- Ich wurde von der unterzeichnenden Studienmitarbeiter mündlich und schriftlich über den Zweck, den Ablauf des Forschungsprojekts, über mögliche Vor- und Nachteile sowie über eventuelle Risiken informiert.
- Ich nehme an diesem Forschungsprojekt freiwillig teil und akzeptiere den Inhalt der zum oben genannten Forschungsprojekt abgegebenen schriftlichen Information. Ich hatte genügend Zeit, meine Entscheidung zu treffen.
- Meine Fragen im Zusammenhang mit der Teilnahme an diesem Forschungsprojekt sind mir beantwortet worden. Ich behalte die schriftliche Information und erhalte eine Kopie meiner schriftlichen Einwilligungserklärung.
- Ich bin einverstanden, dass die zuständigen Fachleute der Projektleitung und der für dieses Forschungsprojekt zuständigen Ethikkommission zu Prüf- und Kontrollzwecken in meine unverschlüsselten Daten Einsicht nehmen dürfen, jedoch unter strikter Einhaltung der Vertraulichkeit.
- Bei Ergebnissen und/oder Zufallsbefunden, die direkt meine Gesundheit betreffen, werde ich informiert. Wenn ich das nicht wünsche, informiere ich den Studienmitarbeiter.
- Ich weiss, dass meine im Rahmen dieser Studie erhobenen Daten nur in verschlüsselter Form zu Forschungszwecken für dieses Forschungsprojekt weitergegeben werden können (auch ins Ausland). Der Sponsor gewährleistet, dass der Datenschutz nach Schweizer Standard eingehalten wird.
- Ich kann jederzeit und ohne Angabe von Gründen von der Teilnahme zurücktreten. Meine weitere Behandlung ist unabhängig von der Teilnahme am Forschungsprojekt gewährleistet. Die bis dahin erhobenen Daten und Proben werden für die Auswertung des Forschungsprojekts noch verwendet.
- Die Institution Schweizer Paraplegiker Forschung haftet für allfällige Schäden.

- Ich bin mir bewusst, dass die in der Informationsschrift genannten Pflichten einzuhalten sind. Im Interesse meiner Gesundheit kann mich die Projektleitung jederzeit ausschliessen.

Ort, Datum	Unterschrift Teilnehmerin/Teilnehmer

Bestätigung der Projektleitung: Hiermit bestätige ich, dass ich dieser Teilnehmerin/diesem Teilnehmer Wesen, Bedeutung und Tragweite des Forschungsprojekts erläutert habe. Ich versichere, alle im Zusammenhang mit diesem Forschungsprojekt stehenden Verpflichtungen gemäss in der Schweiz geltenden Rechts zu erfüllen. Sollte ich im Verlauf des Forschungsprojekts von Aspekten erfahren, welche die Bereitschaft der Teilnehmerin/des Teilnehmers an dem Forschungsprojekt beeinflussen könnten, werde ich sie/ihn umgehend darüber informieren.

Ort, Datum	Name und Vorname Studienmitarbeiter/in in Druckbuchstaben<br><br>Unterschrift Studienmitarbeiter/in

Einwilligungserklärung für Weiterverwendung von Daten in verschlüsselter Form

**BASEC-Nummer (nach Einreichung):**	2024-00087
**Titel des Forschungsprojekts (wissenschaftlich und Laiensprache):**	Druckentlastung und das Auftreten von Dekubitus und Schulterschmerzen: eine prospektive Beobachtungsstudie bei Rollstuhlfahrer*innen mit Querschnittlähmung (wissenschaftlich)<br><br>Zusammenhang von Druckentlastung, Dekubitus und Schulterschmerzen (Laien)
**Teilnehmerin/Teilnehmer:**<br>Name und Vorname in Druckbuchstaben:<br>Geburtsdatum:	

Ich erlaube, dass meine verschlüsselten Daten aus diesem Forschungsprojekt für die medizinische Forschung weiterverwendet werden dürfen. Die Daten werden verschlüsselt gespeichert und für zukünftige, noch nicht näher definierte Forschungsprojekte auf unbestimmte Zeitdauer verwendet.

Ich habe verstanden, dass die Daten verschlüsselt sind und der Schlüssel sicher aufbewahrt wird. Die Daten können im In- und Ausland an andere Datenbanken zur Analyse gesendet werden, wenn diese dieselben Standards wie in der Schweiz einhalten. Alle rechtlichen Vorgaben zum Datenschutz werden eingehalten.

Ich entscheide freiwillig und kann diesen Entscheid zu jedem Zeitpunkt wieder zurücknehmen. Wenn ich zurücktrete, bleiben die Daten verschlüsselt. Dies dient Ihrer medizinischen Sicherheit. Ich informiere lediglich die Projektleitung und muss diesen Entscheid nicht begründen.

Normalerweise werden alle Daten gesamthaft ausgewertet und die Ergebnisse zusammenfassend publiziert. Sollte sich ein für meine Gesundheit wichtiges Ergebnis ergeben, ist es möglich, dass ich kontaktiert werde. Wenn ich das nicht wünsche, teile ich es der Projektleitung mit.

Wenn Ergebnisse aus den Daten kommerzialisiert werden, habe ich keinen Anspruch auf Anteil an der kommerziellen Nutzung.

Ort, Datum	Unterschrift Teilnehmerin/Teilnehmer

Bestätigung der Studienmitarbeiter: Hiermit bestätige ich, dass ich dieser Teilnehmerin/diesem Teilnehmerin Wesen, Bedeutung und Tragweite der Weiterverwendung von Proben und/oder genetischen Daten erläutert habe.

Ort, Datum	Name und Vorname Studienmitarbeiter/in in Druckbuchstaben<br><br>Unterschrift Studienmitarbeiter/in
